# The change of sleeping and lying posture of Japanese black cows after moving into new environment

**DOI:** 10.5713/ajas.18.0048

**Published:** 2018-04-25

**Authors:** Michiru Fukasawa, Tokushi Komatsu, Yumi Higashiyama

**Affiliations:** 1 Graduate School of Agricultural Science, Tohoku University, Osaki, Miyagi, 989-6711, Japan; 2 NARO Tohoku Agricultural Research Center, Morioka, Iwate 020-0198, Japan

**Keywords:** Cattle, Lying, New Environment, Posture, Sleep

## Abstract

**Objective:**

Environmental change is one of the stressful events in livestock production. Change in environment disturbs cow behavior and cows require several days to regain a stable behavioral pattern. Sleeping posture (SP) and lying posture (LP) have been used as indicators for animal that are relaxed and well-acclimated to their environment. The aim of this study was to examine the time required by Japanese black cows for stabilization of SP and LP after moving into new environment.

**Methods:**

Seven pregnant Japanese black cows were used. Cows were moved into new tie-stall shed and their sleeping and lying posture measured 17 times during 35 experimental days. Both SP and LP were detected by accelerometer fixed on middle occipital and hip-cross, respectively. Daily total time, frequency, and average bout of both SP and LP were calculated.

**Results:**

Daily SP time was the shortest on day 1 and increased to the highest on day 3. It then decreased until day 9, after that stabilized about 65 min/d till the end of experiment. Daily LP time changed in same manner as daily SP time. The average SP bout was the longest on day 1, and then decreased to stable level on day 7. On the other hand, the average LP bout was the shortest on day 1, and it increased to stable level on day 7.

**Conclusion:**

These results showed that pregnant Japanese black cows needed 1 week to stabilize their SP. However, there were different change patterns between the average SP and LP bout, even though the change pattern of daily SP and LP time were similar.

## INTRODUCTION

Sleep is an essential behavior for mammals [1]. Many studies suggest the functions of sleep include energy conservation, nervous system recuperation, and emotional regulation [2]. Deep sleep occurs only when the animal is well-accustomed and feels safe in its surroundings [3], as the lack of muscle tone during sleep is innately dangerous for prey animals, such as cattle. Therefore, the sleeping posture (SP), that cattle adopt with their neck relaxed and their head against their flank whilst lying position [2,4], has been used as an indicator that the animal is relaxed and well-accustomed to its environment. Ninomiya and Sato [5] suggested that an enriched environment facilitates SP. Krohn and Munksgaard [6] suggested dairy cows in extensive environments (loose-housing/pasture) slept longer in the SP than when in intensive (tie-stall) environments. Lying posture (LP) has been also used as an indicator of a relaxed and well-acclimated animal [6–8]. Additionally, Oshio et al [7] suggested that daily total time and duration of bout of LP were important indicators of acclimation.

A change in the environment is a stressful event in livestock production. This includes major changes, such as introduction into a new herd after transportation, and minor changes, such as moving to a different stall in same shed. All changes in the environment disturb cow behavior to some extent, which requires several days to stabilize [7,8]. Understanding how cattle acclimatize to new environments could improve productivity and welfare [8]. Therefore, it is necessary to know how long cows take to stabilize SP and LP after moving to a new environment. The aim of this study was to examine the length of time taken for Japanese black cows to stabilize SP and LP after moving to a new environment. Furthermore, we compared between characteristics of SP and LP measurements.

## MATERIALS AND METHODS

This experiment was conducted at NARO Tohoku Agricultural Research Center in January and February 2016. The experimental procedure was approved by the Committee for Animal Experiments of NARO Tohoku Agricultural Research Center (No. 27–6).

### Animals and management

Seven pregnant Japanese black cows were used. Before the experiment, they were kept in a breeding shed at NARO Tohoku Agricultural Research Center. They spent the daytime in a paddock (10:30 to 15:00). At nighttime, they were tethered individually in stanchion stalls with rubber mats (4-cm thick) bedded with sawdust in a shed. Cows were fed grass silage and concentrate in their stalls at 09:00 and 16:00 according to their nutritional requirements [9]. The shed was lit with natural and artificial light during the daytime (09:30 to 17.00) and no artificial light was used during the nighttime. Water and mineral blocks were freely available.

At the start of the experiment, cows were moved into an experimental shed at NARO Tohoku Agricultural Research Center. Some of the cows had prior experience of the shed; however, this was more than 1 year before the experiment. The range of age and parity of the cows at the time of moving into the new shed was 36 to 172-months-old (average 95.7-months-old) and 1 to 10 times (average 5.3 times), respectively. The range of days after conception at the time of moving into the new shed was 59 to 164 days (average 136.9 days). In the new shed, all cows were kept in tie-stalls (175 cm×130 cm) with rubber mats (4-cm thick) bedded with sawdust. They were tethered all day. The barn was lit with natural and artificial light during the daytime (08:30 to 17:00) and no artificial light was used during the nighttime. Average time of sunrise and sunset during the experiment was 06:30 and 17:07, respectively. Cows were fed grass silage and concentrate at 08:30 and 16:00 according to their nutritional requirements. Water and mineral blocks were freely available.

### Measurements

We made a total of 17 measurements during the 35 days of the experiment. Measurements were taken 3 days in a row after cows were moved, and then 3 days each week for 5 weeks. At the start of the experiment, cows were moved into the experimental shed at 10:00. Cow posture was recorded using a video camera and accelerometers, simultaneously. LP, that we defined as cattle grounding body with their legs bent, was recorded using the accelerometer (HOBO-U12-012, onset, Bourne, MA, USA) worn on hip-cross [10]. SP, that we defined as cattle adopt with their neck relaxed and their head against their flank whilst LP, was measured using an accelerometer (HOBO-U12-012, onset, USA) worn on the middle occipital on a halter [4]. Cows were fitted with accelerometers, which recorded their posture from 12:00 to 12:00 the next day. These devices were replaced daily before the morning feed for the first 3 days of the experiment. We recorded the clock time of both the start and end of SP using the accompanying software (HOBOware-Pro, Onset, USA) [4]. The duration of clock times more than 2 minutes was measured. Durations of SP were summed as daily SP time (min/d), and the frequency of SP (times/d) was counted. Averaged SP bout (min/bout) was calculated by dividing daily SP time by the frequency of SP. And daily LP time (min/d), the frequency of LP (times/d), and the average LP bout (min/bout) was calculated as well as SP.

When SP or LP could not be measured using the accelero meter, posture was measured using video. Cow posture was recorded using an infrared camera (WTW-HR872S, Wireless Tsukamoto Co., LTD., Suzuka, Japan) and a digital video recorder (WTW-DH620-2TB, Wireless Tsukamoto Co., LTD., Japan). Four cameras were located at 3.0 m distance around and 2.5 m above the stall. One or two cows were filmed using one camera.

### Statistics

We hypothesized that daily SP time would stabilize after the cattle acclimatized to their new environment. We calculated average daily SP time by shifting the start date from day 1 until day 18. The last date for calculating average daily SP time was fixed on day 35. Subsequently, mean squared deviation (MSD) was calculated for each average daily SP time. To estimate when daily SP time of cows stabilized, we applied two linear regressions to model MSD. The breakpoint observation day was shifted from day 1 to day 18. In the first analysis, MSD was regressed on observation day, and in the second, the regression was set to constant, which included average MSD in the second regression phase. The observation day when the sum of residuals of the two regressions reached minimum was regarded as the inflection day of daily SP time in the new environment.

## RESULTS

**Figure 1:**
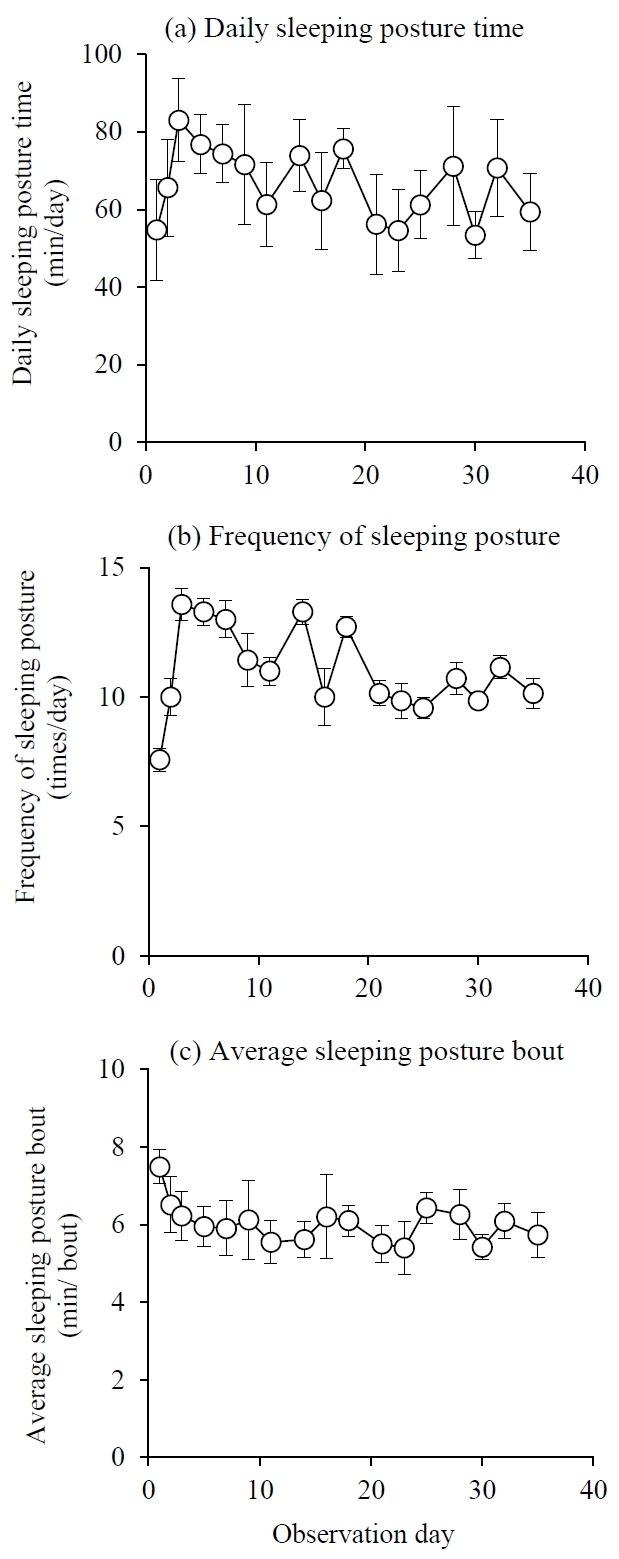
The change of (a) daily sleeping posture time, (b) frequency of sleeping posture and (c) average sleeping posture bout, during experiment. Each symbols and error bar represent average and standard error.

The change in daily SP time after cows were moved to a new environment is shown in Figure 1a. Daily SP time on day 1 was 54.7±12.9 min/d (average±standard error), which was the shortest recorded in the experiment. Daily SP time increased to 83.0± 10.7 min/d on day 3, which was the longest recorded in the experiment, and then decreased until day 9. Thereafter, it fluctuated between 55 and 75 min/d. The change of the frequency of SP after cows were moved is shown in Figure 1b. Cows slept the least number of times on day 1 (7.6±1.9 times/d), but this increased on day 3 (13.6±1.4 times/d), and then decreased until day 9. Thereafter, frequency was about 10 times/d, except for a temporary increase on days 14 and 18. The change in average SP bouts after cows were moved is shown in Figure 1c. The longest SP bout was recorded on day 1 (7.48±0.44 min/bout), and bouts monotonically declined until day 7 (5.90±0.71 min/bout), and then remained stable thereafter.

**Figure 2:**
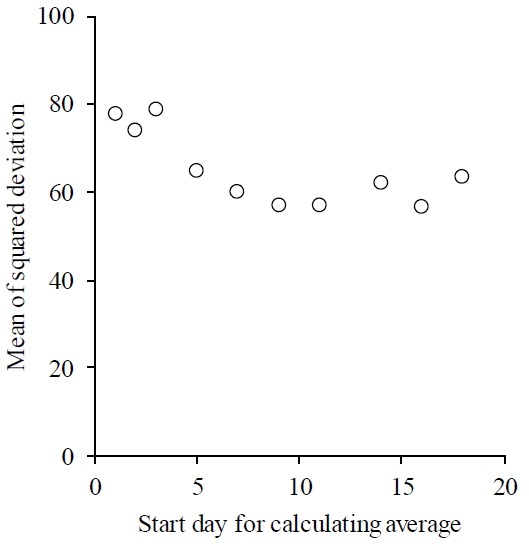
The change of mean of squared deviation between daily sleeping time and average which start date for calculating shifting the start day from day 1 until day 18.

The change in MSD of daily SP time is shown in Figure 2. The highest MSD was observed on day 1, and declined until day 7, except for a temporary increase on day 3. The results of the two regression models are shown in Table 1. The slopes of the first regression were negative, except for the breakpoint set on day 5. The total residual was the lowest on day 5, and the second lowest was on day 7.

**Figure 3:**
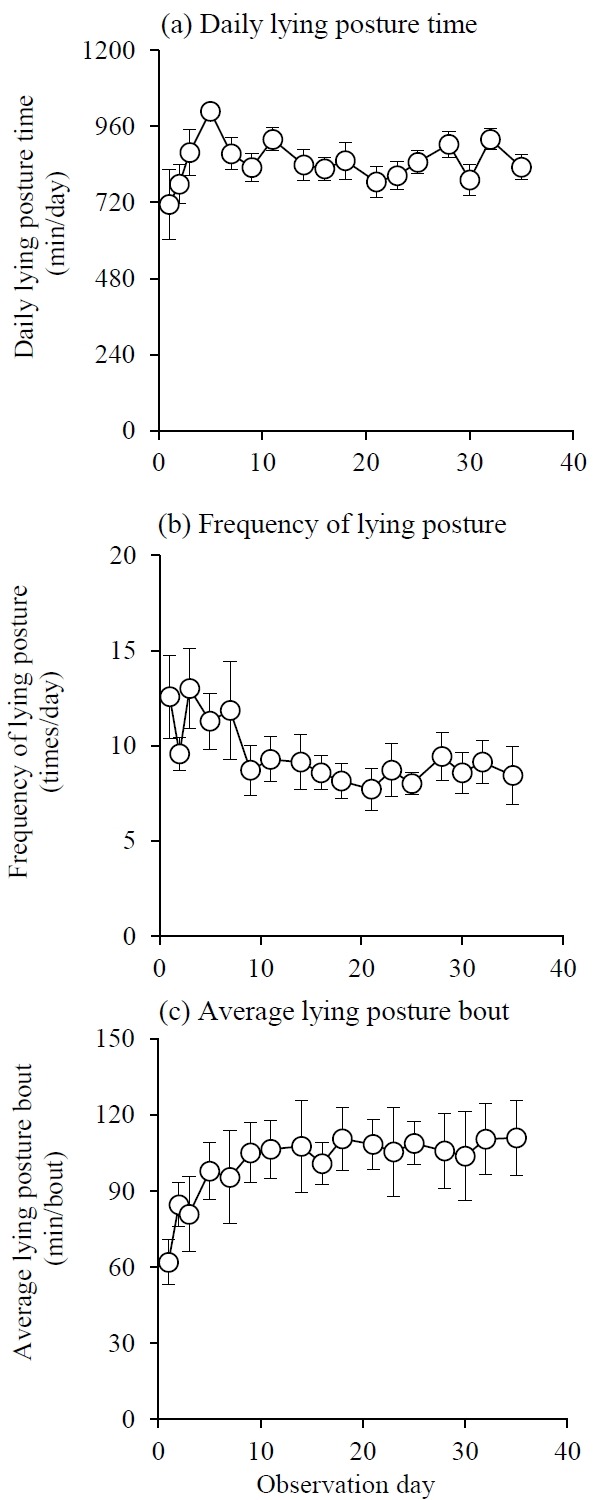
The change of (a) daily lying posture time (b) frequency of lying posture and (c) average lying posture bout during experiment. Each symbols and error bar represent average and standard error.

The change in daily LP time, frequency of LP, and the average LP bout are shown in Figure 3a, 3b, and 3c. Daily LP time tended to change in the same way as daily SP time, reaching a stable level after day 7. The frequency of LP was relatively high from days 1 to 7, and stabilized to about 9 times/d after day 9. The average LP bout was the lowest on day 1 (62.0±8.9 min/bout). Thereafter, it increased and stabilized at around 105 min/bout after day 9.

## DISCUSSION

In this study, total average daily SP time of pregnant Japanese black cows was 66.2 min/d, and ranged from 53.4 to 83.0 min/d. Norring et al [11] reported a similar dairy posture of lying with the neck muscle relaxed, which is equivalent posture to SP in this study (77±12 minutes in multiparous Finnish Ayrshires kept in a tie-stall). Krohn and Munksgaard [6] reported a shorter daily posture of lying with the head turned backwards, which is equivalent posture to SP in this study, (48±4 minutes in loose-housing, and 34±4 min in a tie-stall for monozygotic twins between Danish Friesian and Red Danish). Ninomiya and Sato [7] also reported a shorter daily SP time in Japanese black calves (ca. 20 min in a conventional pen and ca. 40 min in an enriched pen). Since there are many differences in experimental conditions between these studies, e.g., age, sex, breed, tethering, season, it is important to consider how different factors influence the daily SP time of cattle.

The change in daily SP time after cows were moved was too complex to observe using direct regression on the raw data. Therefore, we used the deviation between the observed and assumed stabilized levels, the average of which was calculated by shifting the start date from day 1 to day 18. The total residual was the lowest on day 5; however, the slope of the first regression was positive. This indicated that day 5 was not an appropriate inflection day because the intersection of both regression lines was out of range. Therefore, day 7, which showed the second lowest residual, was used as the appropriate inflection day for daily SP time. Oshio et al [7] reported that heifers and steers required about 1 month to stabilize grazing and rumination patterns after moving. Hasegawa et al [8] reported that behavioral disturbance after regrouping continued for 15 days. In this study, there was no influence of feed stuff, climate change, or social conflict as cows were tethered in a shed and fed the same stuff on almost the same schedule. Although the change in environment in this study was not as drastic as that in earlier studies [7,8], SP and LP were still disturbed by moving the cows, and they took 1 week to stabilize in their new environments. Therefore, attention should focus on cow behavior and posture within the first week after a minor disturbance, such as a change in location in a tie-stall shed, as disturbed cows might suffer a deterioration in productivity and welfare [8].

Both SP and LP have been used as indicators of relaxed and well-acclimated animals [3,5,6]. However, there are some differences between SP and LP. First, deviation of LP between cows in this study was adequate level although the age range of subjected cows was large. On the other hand, deviation of SP between cows was quite large. Therefore, we should use more subjects or consideration of experimental design for using SP as an indicator of cow’s relaxation. Second, the patterns of change were different between the average SP and LP bouts, even though the patterns of daily SP and LP time were similar. Cows spent the shortest time in SP on day 1; however, the average SP bout was the longest. Conversely, both daily LP time and the average LP bout were the shortest on day 1. The average SP bout gradually decreased, and reached a stable level after day 7, while the average LP bout gradually increased, and reached a stable level after day 9. Oshio et al [7] suggested that the duration of grazing and rumination bouts increased as heifers and steers acclimatized to a new grazing environment. Our results supported only the change in average LP bout, but not average SP bout. Krohn and Munksgaard [6] reported that cows laid for longer and slept for a shorter amount of time in tie-stalls, and vice-versa in loose-housing. These results imply a complementary relationship between LP and SP bouts.

SP-related measurements stabilized over 1 week, even after a relatively small change in environment. Careful attention should focus on the complementary relationship between lying and sleeping bouts when these postures are used as behavioral indicators of relaxation or acclimation.

## Figures and Tables

**Table 1:** The detail of two regression model of mean of squared deviation on breakpoint observation day

Breakpoint observation day	1st regression		2nd regression	
	
	Slope	Intercept	Intercept	Total residual
1	-	-	65.1	684.61
2	-	77.7	63.7	500.86
3	−3.92	81.6	62.4	413.14
5	0.44	75.8	60.1	85.56
7	−3.00	82.0	59.3	87.80
9	−3.16	82.3	59.2	89.21
11	−2.86	81.5	59.7	89.34
14	−2.40	80.0	60.7	100.08
16	−1.61	76.8	60.0	192.86
